# In Silico Studies of Novel Vemurafenib Derivatives as BRAF Kinase Inhibitors

**DOI:** 10.3390/molecules28135273

**Published:** 2023-07-07

**Authors:** Teresa Żołek, Adam Mazurek, Ireneusz P. Grudzinski

**Affiliations:** 1 Department of Organic and Physical Chemistry, Faculty of Pharmacy, Medical University of Warsaw, Banacha 1, 02 097 Warsaw, Poland; 2 Department of Toxicology and Food Science, Faculty of Pharmacy, Medical University of Warsaw, Banacha 1, 02 097 Warsaw, Poland

**Keywords:** BRAF kinase inhibitors, vemurafenib derivatives, computational modeling

## Abstract

BRAF inhibitors have improved the treatment of advanced or metastatic melanoma in patients that harbor a BRAF^T1799A^ mutation. Because of new insights into the role of aberrant glycosylation in drug resistance, we designed and studied three novel vemurafenib derivatives possessing pentose-associated aliphatic ligands—methyl-, ethyl-, and isopropyl-ketopentose moieties—as potent BRAF_V600E_ kinase inhibitors. The geometries of these derivatives were optimized using the density functional theory method. Molecular dynamic simulations were performed to find interactions between the ligands and BRAF_V600E_ kinase. Virtual screening was performed to assess the fate of derivatives and their systemic toxicity, genotoxicity, and carcinogenicity. The computational mapping of the studied ligand–BRAF_V600E_ complexes indicated that the central pyrrole and pyridine rings of derivatives were located within the hydrophobic ATP-binding site of the BRAF_V600E_ protein kinase, while the pentose ring and alkyl chains were mainly included in hydrogen bonding interactions. The isopropyl-ketopentose derivative was found to bind the BRAF_V600E_ oncoprotein with more favorable energy interaction than vemurafenib. ADME-TOX in silico studies showed that the derivatives possessed some desirable pharmacokinetic and toxicologic properties. The present results open a new avenue to study the carbohydrate derivatives of vemurafenib as potent BRAF_V600E_ kinase inhibitors to treat melanoma.

## 1. Introduction

The BRAF protein is a serine-threonine kinase, a component of the Ras/Raf/MAPK signaling pathway and one of the best-characterized and globally studied signal transductions from the cell milieu to the cell nucleus, in which, as a result, specific genes are activated for cell growth, division, and differentiation [1]. Signaling through the Ras/Raf/MAPK pathway regulates a variety of cellular functions, such as cell migration and repairs, and also affecting metastasis and angiogenesis processes that are important for tumorigenesis and cancer development [2]. Dysregulation of this pathway is a common event in cancer, as Ras is the most frequently mutated oncogene in human cancer. The mutated BRAF_V600E_ protein leads to constitutive activation of the MAPK signaling pathway, which in turn stimulates growth-factor-independent cellular proliferation and drives oncogenic activity with mitigation of apoptosis and enhanced invasiveness [3]. Identification of BRAF mutations that harbor a BRAF^T1799A^ transversion encoding the constitutively active BRAF_V600E_ oncoprotein substituting the valine at amino acid position 600 prompted several structure-based drug developmental studies that have yielded several highly potent and selective ATP-competitive small molecules used as BRAF inhibitors [4,5,6]. One of the first highly specific BRAF_V600E_ inhibitors, PLX4032 (vemurafenib), was tested in BRIM clinical trials (phase I, II, III) in early 2008 and moved towards rapid approval in the United States (FDA) in August 2011 and the European Union (EMA) in February 2012 [7,8]. Vemurafenib is a low-molecular-weight, ATP-competitive, orally available, highly selective inhibitor of mutated BRAF_V600E_ oncoprotein [9]. This medicine was developed for the treatment of late-stage melanoma to cause apoptosis in melanoma cells by interrupting the BRAF/MEK/ERK pathway only in the presence of the common BRAF_V600E_ mutation. Vemurafenib selectively binds to the ATP-binding site of BRAF_V600E_ kinase and inhibits its activity, which may result in an inhibition of an overactivated MAPK signaling pathway downstream in BRAF_V600E_ kinase-expressing tumor cells and blocks the proliferation of malignant cells that harbor this specific mutation. Studies have shown that vemurafenib does not have activity in wild-type BRAF melanoma cells, unlike other drugs such as sorafenib, which can block both wild-type and mutated BRAF [10].

The pharmacological efficacy of BRAF inhibitors is limited to a subset of cancer patients with BRAF-mutated metastatic melanoma, because the durability of responses in BRAF-mutated melanoma is restricted by the onset of drug resistance mechanisms [11]. The so-called primary resistance to BRAF_V600E_ kinase is mainly supported through the recovery of MAPK/Erk signaling or activation of PI3K/Akt signaling in mutated melanoma. These pathways may be activated through multiple mechanisms, including mutations, copy-number alterations, or changes in gene expression [12]. Note that receptor tyrosine kinases may also act as upstream activators of MAPK/Erk signaling and increase expression in BRAF_V600E_ inhibitor-resistant cells [13]. In addition to upstream activation of MAPK/Erk through receptor tyrosine kinases, increased MAPK/Erk signaling may be also achieved through direct alteration of members of the RAS/RAF/MEK/Erk signaling cascade [14]. Alternative resistance pathways include downstream effectors of PI3K/Akt activation and adaptive resistance through both neighboring cancer and non-cancer cells [15]. Unlike primary resistance, which occurs in approximately 5–10% of patients treated with vemurafenib, the development of secondary resistance to vemurafenib is nearly universal and includes multiple intrinsic and extrinsic pathways [16,17]. Given the multiple reported secondary mechanisms of resistance to BRAF_V600E_ inhibitors, there is an increasing number of ongoing clinical trials investigating various combination regimens of vemurafenib with other molecularly targeted drugs of the MAPK and PI3K/AKT/mTOR pathways [18]. More clinical studies on combinations of vemurafenib with immunotherapy were also recently reported [19,20,21,22].

**Figure 1:**
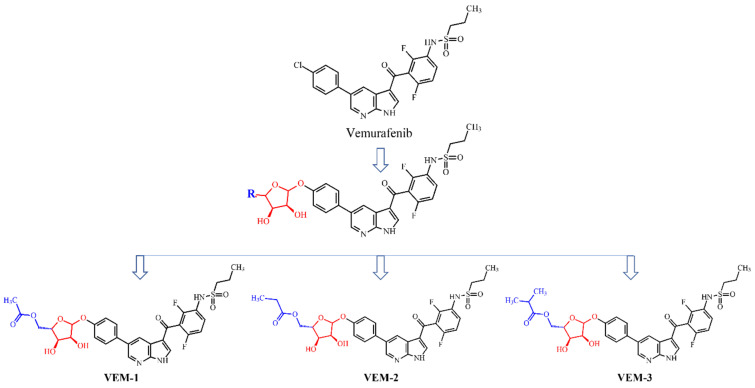
Chemical structures of vemurafenib ((*N*-(3-(5-(4-chlorophenyl)-1*H*-pyrrolo[2,3-b]pyridine-3-carbonyl)-2,4-difluorophenyl)propane-1-sulfonamide) (VEM)) and its three novel derivatives: (5-(4-(3-(2,6-difluoro-3-propylsulfonamido)benzoyl-1*H*-pyrrolo[2,3-b]pyridin-5-yl)phenoxy)-3,4-dihydroxy tetrahydrofuran-2-yl)methyl acetate (VEM-1), 5-(4-(3-(2,6-difluoro-3-propylsulfonamido) benzoyl-1*H*-pyrrolo[2,3-b]pyridin-5-yl)phenoxy)-3,4-dihydroxytetrahydrofuran-2-yl)methyl propionate (VEM-2), and 5-(4-(3-(2,6-difluoro-3-propylsulfonamido)benzoyl-1*H*-pyrrolo[2,3-b]pyridin-5-yl)phenoxy)-3,4-dihydroxytetrahydro furan-2-yl)methyl isobutyrate (VEM-3). (Red color—α-D-ribofuranose; blue color—alkyl groups (methyl, ethyl, and isopropyl)).

The biggest challenge of modern therapies with BRAF_V600E_ inhibitors including vemurafenib is a short time to positive response due to treatments and the multiple drug resistance mechanisms that are developed relatively quickly in melanoma patients. Interestingly, recent findings provided new insights into the role of glycans in mediating melanoma progression [23]. Note that aberrant glycosylation was associated with drug resistance in colorectal cancer, hepatocellular carcinoma and melanoma where sialylation, fucosylation, and N- and I-glycan branching were observed [24,25,26]. Our recent studies on drug resistance mechanisms also indicate that the membrane glycans significantly affect the affinity of vemurafenib to melanoma cells harboring the BRAF_V600E_ mutation [27]. This phenomenon was not observed in non-mutated melanoma cells treated with vemurafenib. Because aberrant glycosylation is scientifically considered a hallmark of cancer as it also occurs in malignant melanoma [28], we aimed to explore other novel vemurafenib derivatives, studying these ligands as candidates for BRAF_V600E_ kinase inhibitors. Here, we report the results from the computational studies of three small molecules with vemurafenib structure conjugated with small pentose-associated aliphatic ligands (Figure 1), focusing on their physicochemical, biopharmaceutical, pharmacokinetic (ADME), and toxicological characteristics and molecular dynamic simulations of these molecules docked to the BRAF_V600E_ kinase receptor, which may be helpful for their optimization or synthesis for further studies. It is expected that the proper modification of vemurafenib will significantly affect the course of its metabolism, and thus contribute to the efficacy and safety profile of new vemurafenib derivatives, leading ultimately to the overcoming of drug resistance.

## 2. Results and Discussion

### 2.1. Drug-Likeness Screening, Biotransformation, and Bioavailability

The topological polar surface area (TPSA) and physiochemical parameters with relation to Lipinski’s rule of five [29] for the analyzed compounds are presented in Table S1 (Supplementary material). In contrast to vemurafenib, the tested derivatives did not match the basic requirements for orally administrated drugs, as the molecular weight (MWt) and TPSA values of these compounds were not found within the range specified for orally administered medicines. Note that the log *p* values are in the range from 3.26 to 3.96 for novel vemurafenib derivatives, which is consistent with a reasonable probability of good absorption (log ≤ 5). For vemurafenib, the log *p* value exceeds 5, indicating a highly lipophilic nature, which may be related to the presence of a chlorine atom. The number of atoms that can be involved in the intermolecular hydrogen bonding for novel vemurafenib derivatives clearly increases such that all vemurafenib derivatives are more susceptible to the formation of hydrogen bonds in the kinase active center than vemurafenib. This was followed by absorption and distribution analysis, considering water solubility (S_w_), effective permeability (P_eff_), apparent permeability (MDCK), the volume of distribution (V_d_), percentage of the unbound drug to blood plasma proteins (%Unbnd), blood-to-plasma concentration ratio (RBP), BBB filter and blood–brain barrier partition coefficient (logBB). The values obtained are presented in Table 1. The aqueous solubility (S_w_ at user-defined pH = 7.4) significantly affects the absorption and distribution characteristics of the compound. Very low S_w_ for novel vemurafenib derivatives is predicted (0.000092–0.00013 mg/mL), which correlates with the values for vemurafenib (0.0000006 mg/mL). Similarly, the parameters in conjunction with the membrane permeability fell within regions representing medium jejunal and MDCK permeation compared to vemurafenib. P_eff_, which reflects passive transport velocity (cm/s) across the epithelial barrier in the human jejunum, is in the region of 0.24–0.34·10^−4^ cm/s and MDCK is in the range of 12.62–14.22·10^−7^ cm/s. The volume of V_d_ is the primary pharmacokinetic parameter that can help define the dose required to give a certain plasma concentration. The V_d_ value of all compounds under study is in the range of 1.02–1.54 L/kg, and these are likely distributed to all tissues in the body.

Another important property of a potential drug candidate is its likelihood of crossing the blood–brain barrier (BBB). The predicted BBB filter showed low values for VEM-1, VEM-2, and VEM-3 as well as vemurafenib, suggesting that these compounds may not easily permeate to the brain, since the computed logBB fell within the recommended range of −3 to 1 [30]. This is a very important result, addressing the problem of increasing the penetration of vemurafenib into brain metastases in aggressive melanoma. Very limited penetration of vemurafenib is expected in the brain tissue due to systemic administration of this agent, and recent studies evidenced ABCB1- and ABCG2-mediated restriction of oral availability and brain penetration of vemurafenib [31]. These results are in agreement with our computer modeling studies on vemurafenib and suggest that the BBB data on vemurafenib derivatives could be considered predictive. The substance’s potency is dependent on the degree of binding to the proteins within blood plasma and whole blood. Two parameters characterizing these properties were tested: the percentage of drug unbound to protein within blood plasma (%Unbnd), and the concentration of the drug in whole blood compared to plasma (RBP). For the tested compounds, the values of %Unbnd are in the range 2.6–4.8%, and the values of RBP are approximately 0.7. This suggests that the tested compounds are highly bound to the proteins within blood plasma.

**Figure 2:**
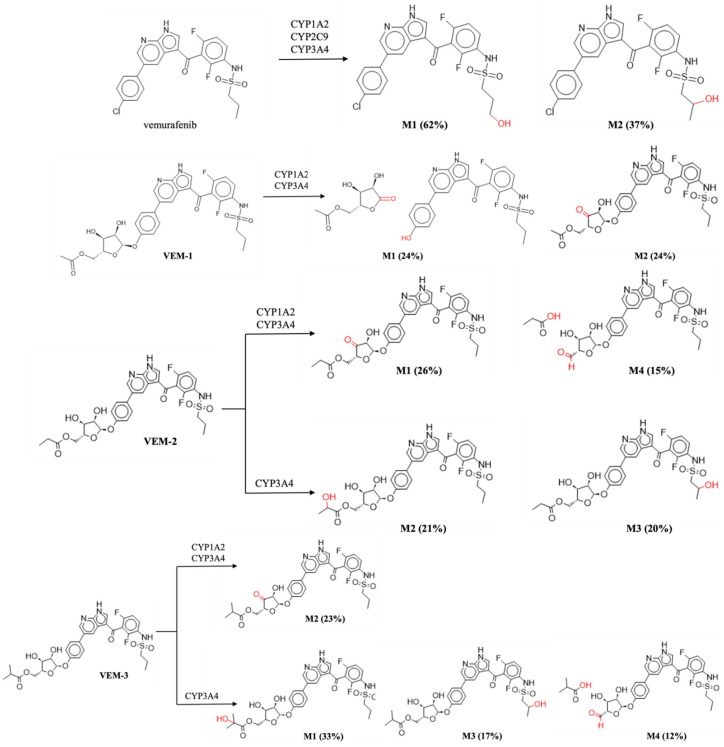
Structures of predicted metabolites for vemurafenib and its derivatives (VEM-1, VEM-2 and VEM-3). Metabolic sites are shown in red color.

Research into the metabolism of drug candidates is fundamental for future studies, since numerous drugs may undergo biotransformation processes to produce several metabolites, which might have different physicochemical, pharmacological, and toxicological properties. In general, the cytochrome P450 performs a significant role in drug metabolism because the primary liver monooxygenase system is involved in phase I biotransformation processes such as oxidation, reduction, hydroxylation, and others. The most significant phenomenon in biotransformation studies is the activation/inhibition of specific cytochrome isoforms [32]. The results obtained for vemurafenib and its derivatives (VEM-1, VEM-2 and VEM-3) are shown in Table S2 (Supplementary material) and Figure 2, elucidating the mode of action on the CYP isoforms and the chemical structure of metabolites. All the selected compounds were found to be substrates and inhibitors of the CYP1A2 isoform, while VEM-2 and VEM-3 may be substrates and inhibitors of both CYP1A2 and CYP3A4. The compounds VEM-1, VEM-2 and VEM-3 cannot be metabolized by the CYP2C9 isoform, while vemurafenib may be its substrate. The tested compounds demonstrated microsomal metabolic liabilities with a wide range of intrinsic clearance (Cl_int_) values ranging from 4.8 to 15.3 μL/min/mg for CYP1A2 and from 76.6 to 1238.95 μL/min/mg for CYP3A4. The highest value (1238.9 μL/min/mg for CYP3A4) was observed for 5-(4-(3-(2,6-difluoro-3-propylsulfonamido)benzoyl-1H-pyrrolo[2,3-*b*]pyridin-5-yl)phenoxy)-3,4-dihydroxy tetrahydrofuran-2-yl)methyl acetal (VEM-1), while the lowest value (C_lint_ < 30 μL/min/mg for CYP1A2) was recorded for compound 5-(4-(3-(2,6-difluoro-3-propylsulfonamido)benzoyl-1H-pyrrolo[2,3-*b*]pyridin-5-yl)phenoxy)-3,4-dihydroxytetrahydro furan-2-yl)methyl isobutyrate (VEM-3) (10.4 μL/min/mg). This could mean that hepatic flow will have minimal influence on its metabolism. In general, the presence of a pentose substituent increases the C_lint_ value compared to vemurafenib. The compounds with C_lint_ > 30 μL/min/mg represent substances that are efficiently removed from intracellular space efficiently via the metabolism and tend to be less available to the systemic circulation. Further analysis predicts metabolic conversion for vemurafenib and its new derivatives, as these compounds show quite an interesting pharmacokinetic profile (Figure 2). The results indicate that CYP3A4 was the major cytochrome responsible for the metabolism of vemurafenib and its derivatives (VEM-1–VEM-3).

The proposed CYP-related biotransformation of vemurafenib in humans leads to the formation of mono-hydroxyl metabolites by hydroxylation occurred at the aliphatic terminus of the sulfonamide chain (up to 62%), which is accordance with the experimental data [33]. In contrast, the predicted metabolites of vemurafenib derivatives resulted in the formation of the post-oxidation products of the sugar fragment, rather than the products of the hydrolysis in the glycosyl bond. In the case of VEM-1 with the present methyl-ketopentose substituent, the action of the enzymatic isoforms CYP1A2 and CYP3A4 leads to the hydrolysis of the glycosidic bond with the release of the carbonyl derivative of the pentose (24%) or the formation of a carbonyl derivative by oxidation of one hydroxyl group in the pentose (24%). The compound of VEM-2 with an ethyl-ketopentose substituent is metabolized by CYP1A2 and CYP3A4 in 26% to carbonyl pentose derivative and the carboxyl derivative with the release of the propanoic acid molecule (15%). Compared to the control (vemurafenib), the VEM-2 derivative also tended to hydroxylate the aliphatic chain and form the primary alcohols via CYP3A4 (20–21%). The VEM-3 compound with isopropyl-ketopentose substituent is prone to conversion to the carbonyl pentose derivative (23%) and hydroxylate the aliphatic chain and form primary alcohols via CYP3A4 (33–17%), similarly to VEM-2. The VEM-3 derivative can form the acid derivative with a carboxyl group in the terminal pentose part (12%) with the liberated methylpropanoic acid molecule. Both metabolites are formed under the influence of the CYP3A4 isoform (Figure 2).

Subsequently, analysis of transport processes was performed by studying the affinities to four major uptake and efflux drug transporters located on the canalicular membrane of hepatocytes, namely, P-glycoprotein (P-gp), breast cancer resistance protein (BCRP), bile salt export pump (BSEP), organic anion transporting polypeptides 1B (OATP1B1 and OATP1B3), hepatic organic cation transporters (OCT1 and OCT2), and renal organic cation transporters (OAT1 and OAT3). The values of the parameters are presented in Table 2 and Table S3 in Supplementary material. It was important to determine whether the newly designed compounds are inhibitors or substrates of drug transporters. Multidrug efflux transporters of the ATP-binding cassette (ABC) protein family, such as P-gp and BCRP, can have important roles in the efficacy of chemotherapy.

Consequently, they affect oral uptake and tissue distribution of their wide range of substrate drugs, for example, by restricting their intestinal uptake, thus reducing oral bioavailability, and by restricting their brain penetration. As shown in Table 2, the compounds (VEM-1, VEM-2, VEM-3), unlike vemurafenib, can be predicted as both substrates and inhibitors of P-gp and BCRP, and thus have the potential to affect the drug resistance mechanism of cancer. It was found that the compounds (VEM-1, VEM-2, VEM-3) have the potential to inhibit OATP1B1 and OATP1B3, and they have been reported as BSEP inhibitors. In turn, OAT1 and OAT3 catalyze uptake across the basolateral membranes of kidney cells, where they are responsible for removing their substrates from general circulation. Our study shows that the tested compounds were found to be potent inhibitors of both transporters (see Table S3 in Supplementary material). Note that OCTs refer to one of the most abundant transporters of the liver and they are considered poly-specific membrane transporters that act by mediating the hepatic uptake of hydrophilic compounds that are small and positively charged. It has been suggested that all the tested compounds would act as inhibitors for OCT1 and OCT2.

### 2.2. Simulation of Plasma Concentration–Time Profile in Humans

Initially, to validate the predicted plasma concentration–time profile (Cp–time profile) for the new compounds (VEM-1–VEM-3), we performed a simulation of vemurafenib based on the original study by Zhang et al. [34]. This study characterized the multiple-dose pharmacokinetics of vemurafenib 240 mg, 480 mg, 720 mg and 960 mg twice daily (bid) in BRAF_V600E_ mutation-positive metastatic melanoma patients, using the commercial formulation (240 mg micro-precipitated bulk powder film-coated tablets). Based on the selected input dataset, we used an appropriate model to predict healthy human subjects’ Cp–time profile after single oral administration of 240 mg, 480 mg and 960 mg of vemurafenib. In all simulations, a default simulation time of 8 h and 24 h was used, using an IR tablet as the dosage form. The simulated Cp–time profiles of vemurafenib and its three derivatives (VEM-1–VEM-3) are shown in Figure S1 in Supplementary material. The relevant summary statistics (C_max_, T_max_, AUC_0–8h_, AUC_0–24h_ and t_1/2_) are compared in Tables S4 and S5 in Supplementary material. The obtained experimental values show that vemurafenib exposure (AUC and C_max_) is dose-proportional over the 240 to 960 mg bid dose range and exhibits constant drug levels over the bid dosing interval. Vemurafenib is absorbed rapidly after a single oral dose of 960 mg, reaching maximum drug concentration approximately 5 h after administration, and exhibited a mean terminal phase half-life of 31.5–38.4 h. The predicted results were consistent with clinically observed data: with once-daily dosing, exposure levels (C_max_, AUC_0–8h_ and AUC_0–24h_) also increased with increasing dose [34].

In the next step, the bioavailability profile for the VEM-1, VEM-2 and VEM-3 derivatives were assessed in relation to vemurafenib (Table S5 in Supplementary material). It can be seen that all simulated parameters for the tested compounds showed significant variability compared to vemurafenib. The simulated AUC_0–24h_ for the 240 mg dose with VEM-1 was 230 ng-h/mL, and AUC_0–8h_ and C_max 0–8h_ ratio 80 ng-h/mL and 13.9 ng/mL, respectively. Thus, the high accumulation of the compound VEM-1 already occurs at the lower dose given once daily compared to vemurafenib. VEM-1 administered once daily at a dose of 240 mg is absorbed with a median T_max_ of approximately 6 h. Increasing the dose of VEM-1 from 240 mg to 960 mg once daily effectively proportionally increased the plasma concentration of VEM-1 at the same T_max_. In the simulation of 960 mg of VEM-1, both the AUC_0–8h_ and C_max 0–8h_ of VEM-1 increased to 135.4 ng-h/mL and 23.6 ng/mL, respectively. In addition, it showed a mean terminal plasma half-life in the range of 13.8–14.0 h, so the degree of accumulation is significantly long at such a low administered dose. The simulated AUC from 0 to 8 h and C_max_ values for VEM-2 and VEM-3 at doses of 240–960 mg administered once daily were lower than VEM-1. The medians of the geometric mean value C_max 0–8h_ and AUC_0–8h_ were 9.67 ng/mL and 54.3 ng-h/mL (VEM-2), respectively, and for VEM-3 C_max 0–8h_ 4.2 ng/mL and AUC_0–8h_ 23.7 ng-h/mL at a once-daily dose of 960 mg. It is predicted that VEM-2 and VEM-3 will be absorbed rapidly after a single oral dose of 960 mg, reaching maximum compound concentration approximately 6 h after administration. Extensive accumulation should occur after multiple dosing at 960 mg twice daily, similarly to vemurafenib.

### 2.3. Toxicity Assessment

For any compound to be considered a potential drug candidate in clinical trials, it should have an acceptable pharmacokinetic profile, as well as a high safety margin with a lower probability of toxicity and potent adverse effects. More details on regulatory affairs are well described in EMA, FDA and ICH guidelines. The toxicology profile of the tested compounds was evaluated using both animal and human toxicology (in silico) approaches (Table 3 and Table 4). All the tested compounds have threshold of the maximum recommended therapeutic (human) dose (MRTD) values below 3.16 mg/kg/day. The calculated likelihood of the cardiotoxic effects of vemurafenib and its derivatives (VEM-1, VEM-2, VEM-3) was assessed as non-toxic based on the data on the hERG pIC_50_ value as a measure of affinity for the hERG K^+^ channel. Parameters related to liver toxicity are also found to be acceptable (Table 3). Vemurafenib and its new derivatives are not expected to cause hepatotoxicity, which would likely be the cause of drug-induced liver injury (DILI).

The toxicity and genotoxicity of the tested compounds were then estimated computing toxic/genotoxic endpoints based on animal models, such as those reported for acute toxicity (rat acute toxicity Rat LD_50_) and chronic toxicity (carcinogenicity) in rodents (rat TD_50_ and mouse TD_50_). The results of computation studies for genotoxicity including in silico the AMES assay and clastogenicity studies based on chromosomal aberration assessment (Chrom Aberr) are shown in Table 4.

Two carcinogenicity models estimating the TD_50_ value (mg/kg/day), where the TD_50_ is the oral daily dose administered over the course of a lifetime required to produce tumors in 50% of the population, were determined. The predicted carcinogenicity TD_50_ values for the mouse model were above 600 mg/kg/day for all compounds tested; hence, it can be concluded that none of the tested compounds induced tumors in mice. In turn, the prediction of the median chronic toxic dose for rats (rat TD_50_) was lower for vemurafenib derivatives (1.38–1.99 mg/kg/day) than the parent drug (21.93 mg/kg/day). Regarding the carcinogenicity model in rats, vemurafenib derivatives were considered to elucidate some carcinogenic potentials. This was not observed for vemurafenib or its derivatives tested for TD_50_ in mice (Table 4). In the LD_50_ (lethal dose) model in rats by oral ingestion, the predicted LD_50_ ranged from 44 mg/kg b.w. to 156.29 mg/kg b.w. Thus, vemurafenib was considered to be the most toxic agent based on the median lethal dose. These results will be helpful in setting the dose ranges for in vivo preclinical studies using rats and mice. No potential risk of clastogenic activities based on chromosomal aberrations was observed for any of the tested compounds. In terms of vemurafenib and its derivatives, the predicted mutagenicity responses varied against different strains of *Salmonella typhimurium* (Table 4). Note that vemurafenib and its derivatives VEM-2 and VEM-3 show predictable mutagenicity in only *Salmonella typhimurium* strains TA97 and/or TA1537, while predictable mutagenicity in other strains is not observed.

The toxicity risk model of the tested compounds is estimated based on the three risk computation models: MUT_Risk_, MUT_xRisk_ and TOX_Risk_ (see Table S6 in Supplementary material). Predicted toxicity for the compounds is relatively low; however, the individual data may also consider the risk of chronic/carcinogenic potential based on rat studies and petite mutagenicity in *Salmonella typhimurium* in some tests with post-mitochondrial fraction S9. This could be suggested for some metabolite-associated effects, if any. That computation did not predict MUT_Risk_ and MUT_xRisk_ for compound VEM-1. Generally, the predicted ADME-TOX properties for novel vemurafenib derivatives suggest that these molecules elucidate some desirable pharmacokinetic and toxicologic properties. Therefore, we decided to use a computational approach for the initial screening of their potency targeting the BRAF_V600E_ kinase protein as a potent inhibitor.

### 2.4. Binding Site in the BRAF_V600E_ Kinase

In order to better understand the potency of the proposed new vemurafenib derivatives (Figure 1), we proceeded to study their interaction with the BRAF_V600E_ kinase oncogenic mutant crystal structure (PDB code: 3OG7). The receptor 3OG7, which was co-crystalized with PLX4032 (vemurafenib), was used as the molecular target, and vemurafenib was treated as a reference inhibitor for the testing of binding affinities to the BRAF_V600E_ [35]. From the crystal structure of 3OG7, it is known that the active site of BRAF_V600E_ is formed from residues such as Ile-463, Gly-464 and Val-471 of P-loop, Leu-505 and Thr-508 of αC-helix, Arg-575, Asp-576, Lys-578, Asn-580, and Asn-581 of the catalytic loop, Asp-594, Phe-595, Gly-596 of DGF motif, Ala-481, Lys-483, Ile-513, Leu-514, Phe-516, Ile-527, Thr-529, Gln-530, Trp-531, Cys-532, Gly-534, Ser-535, Ser-536 and His-539 of N-lobe, Phe-583, Gly-593, Glu-600, Lys-601, Arg-603, Tpr-604 and Ser-605 of C-lobe. From the above residues, Val-471, Ala-481, Lys-483, Leu-514, Thr-529, Trp-531, and Cys-532 are key residues interacting with a ligand and Asp-594, Phe-595 and Gly-596 compared to a DGF motif, which determined the inactive or active state of BRAF protein. The described pocket was used in the molecular docking procedure employing the CDOCKER module accessible in DiscoveryStudio v.21.1 to define the starting conformations of ligands (vemurafenib, VEM-1, VEM-2 and VEM-3) for the molecular dynamics (MD).

The re-docking of vemurafenib at the ATP-binding site of the BRAF_V600E_ kinase active region was preliminarily carried out to validate the reference ligand (vemurafenib) was docked to the kinase in the way in which it was experimentally observed. The root-mean-square deviation (RMSD) between the docked and crystal structure of vemurafenib was only 0.948 Å (less than 1 Å). Figure S2A in Supplementary material shows that the docked structure (yellow) and X-ray crystal structure (green) are quite similar. The resulting complex showed eleven residues participating in the intermolecular interactions—Val-471, Ala-481, Lys-483, Leu-505, Leu-514, Thr-529, Trp-531, Cys-532, Asp-594, Phe-595, and Gly-596—which are essential for the activity of these class inhibitors (Figure S2B,C in Supplementary material). Then, the compounds (VEM-1, VEM-2, VEM-3) were docked to the ATP-binding pocket of the BRAF_V600E_ protein. The lowest docking pose energy was selected as the docking score with the best protein affinity. The energies of the best pose, estimated by intermolecular energy, including van der Waals energy, hydrogen bonding energy, and electrostatic energy, ranged from −57.46 kcal/mol to −68.82 kcal/mol. When the CDOCKER interaction energy was compared, the VEM-3 derivative (−68.82 kcal/mol) demonstrated the lowest CDOCKER interaction energy, lower than vemurafenib (−63.91 kcal/mol), while VEM-1 (−57.72 kcal/mol) and VEM-2 (−57.46 kcal/mol) showed comparable CDOCKER interaction energy. The docking result was deemed not to be completely consistent with an inhibitory activity. Therefore, to further investigate the interaction between the BRAF_V600E_ kinase and vemurafenib derivatives, molecular dynamic (MD) simulations were carried out in the subsequent stage.

### 2.5. Binding Free Energy and Intermolecular Interactions in the BRAF_V600E_ Binding Pocket

**Figure 3:**
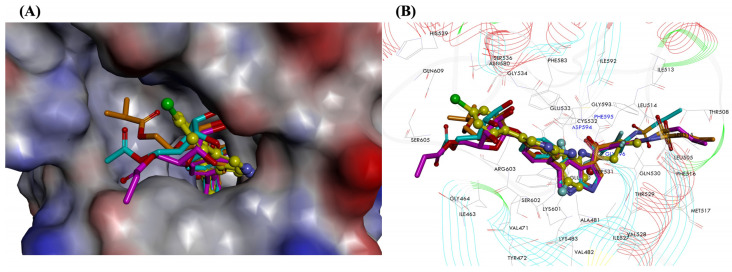
Closeup view of an active site of the BRAF_V600E_ kinase structure after MD simulations. Surface hydrophobicity was depicted by the shaded colors: brown—the hydrophobic and blue—the hydrophilic regions. (**A**,**B**) Predicted superposition of compounds: vemurafenib (C atoms shown as yellow), VEM-1 (C atoms shown as turquoise), VEM-2 (C atoms shown as pink), and VEM-3 (C atoms shown as orange).

MD simulations were performed for each of the BRAF_V600E_—ligand complexes at the ATP-binding site. The RMSD values between the initial ligand structures and the results from average structures were low in all cases. The binding free energy (ΔG_bind_) values for the tested compounds were calculated using the MM-PBSA approach. The calculated strength of interactions with BRAF_V600E_ kinase decrease in the sequence: VEM-3 > VEM-1 > VEM-2 (−90.98 kcal/mol > −64.96 kcal/mol > −38.32 kcal/mol, respectively). For the BRAF_V600E_ kinase–vemurafenib complex, the ΔG_bind_ value is equal to −81.71 kcal/mol. The obtained results showed that all the vemurafenib derivatives are effective in targeting BRAF_V600E_, especially the VEM-3 derivative, with binding energy better than that of vemurafenib, the standard BRAF_V600E_ inhibitor. The resulting MD orientations of the tested compounds in the active kinase domain of BRAF_V600E_ are presented in Figure 3. The intermolecular distance, which is responsible for key interaction with the active site of the BRAF_V600E_ kinase structure after MD simulations, is shown in Table S7 (see Supplementary Materials).

**Figure 4:**
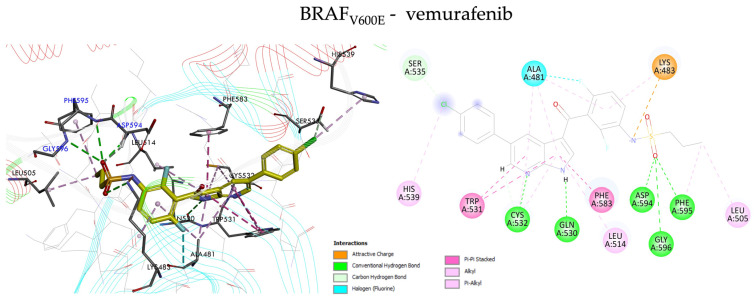
Predicted binding mode of BRAF_V600E_ inhibitor vemurafenib. Significant intermolecular interactions are indicated with dashed lines.

**Figure 5:**
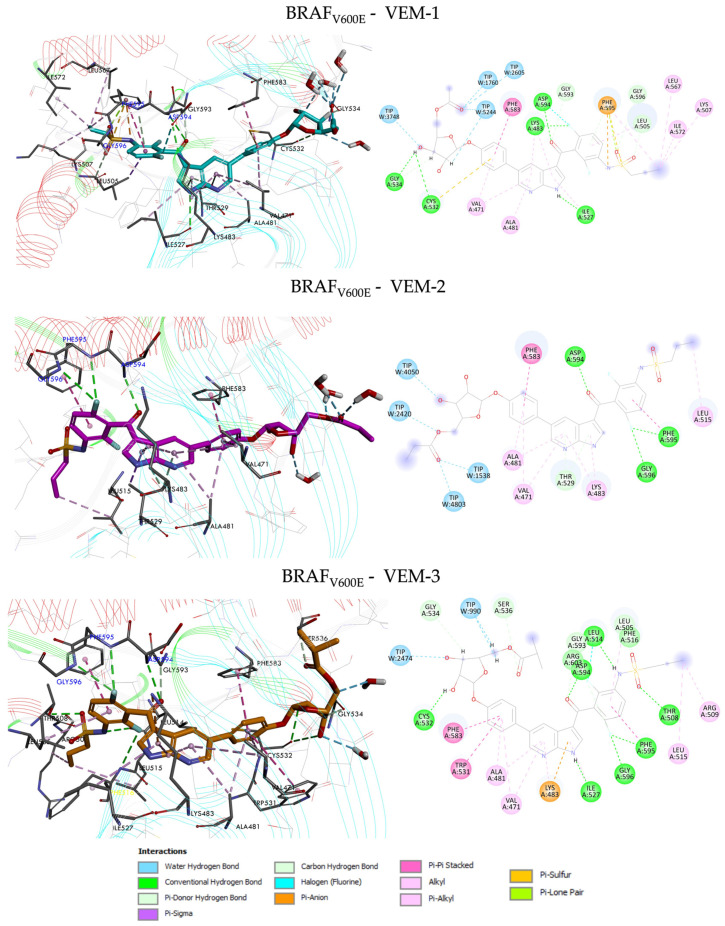
Predicted binding mode for VEM-1, VEM-2, and VEM-3 derivatives with BRAF_V600E_. Significant intermolecular interactions are indicated with dashed lines.

The comparison of position and orientation of the tested compounds (VEM-1, VEM-2, VEM-3) with vemurafenib used as the reference inhibitor exhibited a slight difference in binding mode, indicating a significant movement of kinase and ligands during the MD process. The central pyrrole and pyridine rings of novel vemurafenib derivatives were similarly located within the hydrophobic ATP-binding site of BRAF_V600E_ and mainly surrounded by Val-471, Ala-481, Lys-483, Ile-527 and Thr-529. The remaining molecule elements (sulfonamide group, pentose ring and alkyl chains) were the deciding factors in showcasing the differences in binding modes with BRAF_V600E_ kinase. The detailed types of interactions involved in the designed compounds VEM-1–VEM-3 and vemurafenib are presented in Figure 4 and Figure 5.

The azaindole core of vemurafenib forms two strong hydrogen bonds with the residues Gln-530 and Cys-532 and hydrophobic interactions with Leu-505, Ala-481, Leu-514, Trp-531 and Phe-583 in the hinge region. The *para*-chloro substituent of the phenyl protrudes out of the ATP-binding pocket forming a carbon–hydrogen bond with Ser-535 and hydrophobic interaction with His-539 (Figure 4). On the other side of the molecule, the sulfonamide moiety interacts with the DFG motif of BRAF_V600E_, forming a strong hydrogen bond between the N-H of Asp-594, Phe-595 and Gly-596, and the electrostatic interaction of sulfonamide nitrogen atom with the Lys-483, whereas the terminal aliphatic propyl group occupies a lipophilic back pocket. In addition, one halogen bond between the fluor atom of the difluoro-phenyl of vemurafenib and Ala-481 residue can be observed.

The VEM-2–BRAF_V600E_ complex shows the lowest binding free energy, which indicates the possibility of fewer stable intermolecular interactions between the ligand and the protein target than the complexes of VEM-1–BRAF_V600E_ and VEM-3–BRAF_V600E_, respectively. The compound VEM-2 was bound to the active site of the protein with similar residue involvement to vemurafenib (Figure 5). The interactions of the compound VEM-2 are mainly with residues Asp-594, Phe-595 and Gly-596 from the DGF loop. The other interactions that stabilize the formation of this complex are noticeably weaker with residues (π–alkyl interactions associated with Val-471, Ala-481, Lys-483 and Leu-515, π–donor hydrogen bond interaction associated with Thr-529, and π–π stacked interactions associated with Phe-583 and Phe-595). No key interactions were detected with residues Leu-514, Trp-531, or Cys-532 from the hinge loop. Moreover, the position of the ethyl-ketopentose substituent of VEM-2 allows for creating interactions with only four water molecules. Two complexes, VEM-1–BRAF_V600E_ and VEM-3–BRAF_V600E_, marked by high binding energy, are stabilized by much more intermolecular interactions. The compound VEM-3 docked inside the active site of BRAF_V600E_ kinase revealed a better score than vemurafenib, showing that it has bound in the active site through the formation of seven conventional and five carbon–hydrogen bonds with residues Leu-505, Thr-508, Leu-514, Ile-527, Cys-532, Gly-534, Ser-536, Gly-593, Asp-594, Phe-595, Gly596, and Arg-603, which are essential for the activity of the classic inhibitors. The nitrogen atom of the sulfonamide group and fluor atoms of the aminobenzene ring formed strong hydrogen bonds with Leu-514, Gly-593, Gly-596, Arg-603, and Phe-595. The next was formed between the oxygen atoms of the sulfonamide moiety and carbonyl group to residues Thr-508, Leu-505, Gly-593, and Asp-594. One strong hydrogen bond was also observed between the nitrogen atom of the pyrrole ring with Ile-527 residue, and the other one was formed between the hydroxyl group of the pentose ring to Cys-532 from the hinge loop.

Additionally, the isopropyl-ketopentose substituent of VEM-3 created the next hydrogen bond with Gly-534, Ser-536 and two water molecules. There were two arene π–π interactions within the binding site and the ligand with Trp-531, Phe-583 and Phe-595, which have occurred due to the intercalation of the benzene ring. The stability of the complex might be also associated with extra alkyl interaction with Arg-509 and Leu-515, π–alkyl interaction with four residues (Val-471, Ala-481, Lys-483 and Leu-505), and lastly π–cation interaction between the pyrrole ring of VEM-3 and Lys-483 residue. Therefore, we can assume that the VEM-3 derivative may inhibit the growth of melanoma cells through the inhibition of the BRAF_V600E_ kinase in the same manner as vemurafenib.

Similarly, to the compound VEM-3, the binding mode of the optimized structure of the compound VEM-1 with the present methyl-ketopentose substituent showed that most of the interactions are of a hydrogen bond type with the same residues (Leu-505, Ile-527, Cys-532, Gly-534, Gly-593, Asp-594 and Gly-596). While residues that participated in the anchoring of both compounds are almost the same, there are significant differences in quantitative terms, which can explain why compound VEM-1 is a little weaker as an inhibitor than compound VEM-3. As shown in Figure 5, we can observe differences in the interactions compound VEM-1 with key residue Phe-595 from the DFG loop and Cys-532 from the hinge loop. The nitrogen and sulfur atoms of the sulfonamide moiety formed one π–anion interaction and one π–sulfur interaction with Phe-595 residue. Moreover, the π–sulfur interaction was established between a ring (benzene) and Cys-532 residue. Noteworthily, the position of the methyl-ketopentose substituent of VEM-1 allows for creating interactions with four water molecules.

From the obtained results, we can observe that H-bonding is the main force controlling the interactions that exist between the docked compounds (VEM-1, VEM-2, VEM-3) and the BRAF_V600E_ protein and that the interaction energy of the compounds increases with the increase in the number of the hydrogen bonds. It could be observed that in the conventional hydrogen bonding identified with the designed derivatives, the number of residues that participated was found to be better than vemurafenib, as shown in Figure 5 and Table S7 in Supplementary Materials, and there were high similarities. Noteworthily, some of the designed vemurafenib derivatives interacted more with the target residues through electrostatic forces, especially compounds VEM-1 and VEM-3.

### 2.6. Stability Assessment of MD Simulations

**Figure 6:**
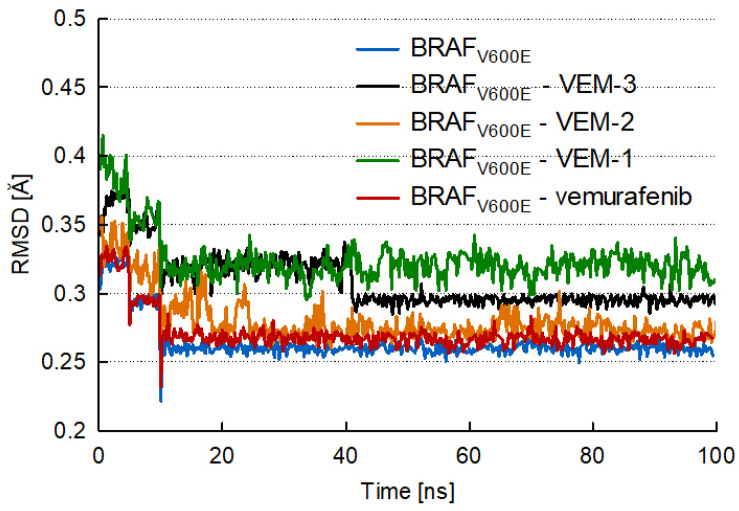
RMSD *versus* time for the BRAF_V600E_ protein, vemurafenib, VEM-1, VEM-2, and VEM-3 derivatives with BRAF_V600E_.

To evaluate the stability of the MD simulations, the properties of each BRAF_V600E_–ligand complex were inspected during the entire MD trajectory using root-mean-square deviation (RMSD) and root-mean-square fluctuation (RMSF) analysis. The trajectory of the RMSD and the RMSF of the protein–ligand complex was monitored using the Analyze Trajectory tool of DiscoveryStudio v.21.1. software. The RMSD determines the stability of the protein–ligand complex by assessing the equilibration period of the MD simulation and denoting the dynamic changes in protein and ligand at a specific temperature during the simulation [36]. RMSD values at 300 K over the course of the 100 ns were outlined for the trajectory structures of all studied complexes (Figure 6).

As shown in Figure 6, the RMSDs for the ligand-bond BRAF_V600E_ kinase obtained for a 100 ns simulation indicated that stable complexes were formed. The RMSD of each complex initially did not exhibit robust stabilization. It was only after 10 ns that they maintained steady-state stability throughout the next simulation period for up to 100 ns. Only a slight bump was observed for the BRAF_V600E_–VEM-2 complex in the range of 10–25 ns. The results revealed that the RMSD of each system tended to converge after 40 ns simulation, indicating that the systems were stable and equilibrated. The BRAF_V600E_ kinase with VEM-1 showed RMSD fluctuation from 0.3 to 0.35 Å, while for the other three complexes (BRAF_V600E_–VEM, BRAF_V600E_–VEM-2, and BRAF_V600E_–VEM-3), RMSD varied from 0.25 to 0.3 Å with no major deviation. All complexes revealed a good level of interaction throughout the simulation, with less deviation in structure.

**Figure 7:**
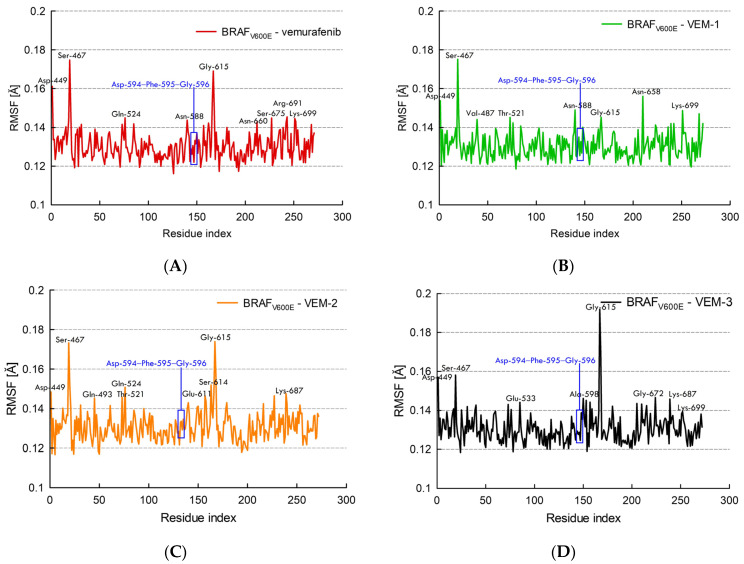
RMSF of protein complexes (**A**) vemurafenib–BRAF_V600E_ kinase, (**B**) VEM-1–BRAF_V600E_ kinase, (**C**) VEM-2–BRAF_V600E_ kinase, and (**D**) VEM-3–BRAF_V600E_ kinase.

To investigate the change in flexibility of each residue during ligand (vemurafenib, VEM-1, VEM-2 and VEM-3) binding with BRAF_V600E_, the RMSF value of each residue, which represents the deviation of the position from a reference position during the whole simulation period, was calculated and is displayed in Figure 7.

The larger the range of fluctuations and/or the number of residues engaged in fluctuation, the worse the ligand and protein binding, and the more likely the ligand and protein will dissociate from the binding site throughout the MD simulation run time. In all complexes, the RMSF for each residue surrounding the ligand is lower than 0.2 Å, which shows that the binding pocket is quite stable during the MD simulation. The RMSF values of all ligand–BRAF_V600E_ kinase complexes were found to be distributed between 0.12 Å and 0.14 Å with mild fluctuation. The highest RMSF values were for Ser-467 and Gly-615, residues whose side chain pointed toward the solvent, far from the binding site of the ligand, except for VEM-1, which showed the highest fluctuation at the Asn-658 position in addition to Ser-467. The intermediate residues that undergo slightly smaller fluctuations depending on the ligand included Thr-521, Gln-524, Asn-588, Lys-699, Lys-687 and Ser-675. It is important to note that the catalytically relevant residues Asp-594, Phe-595 and Gly-596 have RMSF values below 0.14 Å.

### 2.7. Chemical Reactivity of VEM-3

**Figure 8:**
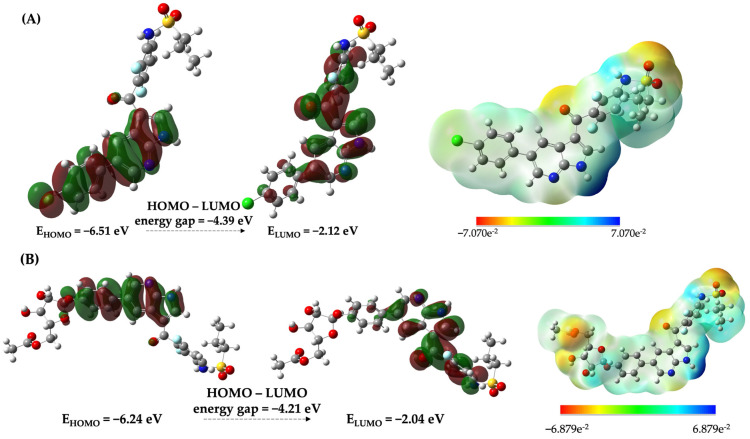
Visualization of HOMO, LUMO, and molecular electrostatic potential (MEP) surfaces for (**A**) vemurafenib and (**B**) VEM-3, calculated at the B3LYP/6-311G (d,p) level of theory. The positive (red) and negative (green) phase distributions in molecular orbital wave function. The MEP represents electron-rich (red), electron-poor (blue) and neutral potential (green) regions.

The structural and electronic properties of the best compound (VEM-3) were calculated through density functional theory (DFT) studies to explore the sites of chemical reactivity, which were used to identify the possible regions of the interaction of potential inhibitors with the BRAF_V600E_ kinase. Electronic properties such as the frontier molecular orbitals (FMOs) and molecular electronic potential (MEP) maps were analyzed (Figure 8). The FMOs are relevant for predicting the relative reactivity based on the electronic structure properties of a molecular system. The chemical properties of a molecule are controlled by the valence orbitals. In this way, the nucleophilic attacks are controlled by the HOMO orbital, and the electrophilic attacks are controlled by the LUMO orbital. The HOMO–LUMO gap energy is considered a measure of molecular structure stability, whereas the MEP is a suitable parameter to define the regions of a molecule susceptible to electrophilic or nucleophilic interaction with the surface of the kinase. In Figure 8, the atoms with positive electrostatic potentials are depicted in blue and the regions carrying negative electrostatic potentials are displayed in red.

Examining the shapes of frontier MOs for the reference inhibitor vemurafenib, it is noted that the HOMO exhibits a delocalized shape that is extended over a conjugated π–system including the pyridine–pyrrole moiety, whereas the LUMO is extended over most parts of the molecule including the fluorine-substituted benzene ring and very close to the sulfonamide group. The calculations revealed an energy gap (HOMO–LUMO) of −4.39 eV. The formation of VEM-3 did not induce a substantial change in the nature of the HOMO and LUMO; however, it is worth noting that the energy gap was slightly affected, with a value of −4.21 eV, which may indicate its higher reactivity.

Figure 8 shows the MEP map in a range between −7.07e^−2^ and 7.07e^−2^ a.u. for the vemurafenib and −6.88e^−2^ and 6.88e^−2^ a.u. for VEM-3 using an isovalue of 0.0004 a.u. In both compounds, hydrogen atoms present in the pyrrole moiety and sulfonamide group show intense blue regions, have the highest positive potential, and are responsible for nucleophilic attacks on surrounding groups, whereas all oxygen atoms of the carbonyl and sulfone groups have a red region showing the highest negative potential and are responsible for electrophilic attacks in those compounds. For VEM-3, we can additionally note that the negative electrostatic potentials are located around the oxygen atoms coming from the isopropyl-ketopentose moiety. Thus, these regions have high electron density and could interact with the electrophilic groups of BRAF_V600E_ kinase, as confirmed by MD analysis.

## 3. Materials and Methods

### 3.1. Starting Structure Preparation and Modeling

Four compounds were tested: vemurafenib (*N*-(3-(5-(4-chlorophenyl)-1*H*-pyrrolo[2,3-b]pyridine-3-carbonyl)-2,4-difluorophenyl)propane-1-sulfonamide) (VEM) and its three as-designed novel derivatives 5-(4-(3-(2,6-difluoro-3-propylsulfonamido)benzoyl-1*H*-pyrrolo[2,3-b]pyridin-5-yl)phenoxy)-3,4-dihydroxy tetrahydrofuran-2-yl)methyl acetate (VEM-1), 5-(4-(3-(2,6-difluoro-3-propylsulfonamido) benzoyl-1*H*-pyrrolo[2,3-b]pyridin-5-yl)phenoxy)-3,4-dihydroxytetrahydrofuran-2-yl)methyl propionate (VEM-2) and 5-(4-(3-(2,6-difluoro-3-propylsulfonamido)benzoyl-1*H*-pyrrolo[2,3-b]pyridin-5-yl)phenoxy)-3,4-dihydroxytetrahydro furan-2-yl)methyl isobutyrate (VEM-3) (Figure 1). Three-dimensional structures of the ligands were prepared using DiscoveryStudio v.21.1 visual interface BIOVIA [37]. The starting conformations of three newly designed derivatives—VEM-1, VEM-2, and VEM-3—were constructed based on the solid-state diffraction data of vemurafenib to eliminate any subjectivity in generating the three-dimensional structures. In the first stage of the study, the conformational space was found for each compound to determine the most stable, lowest-energy conformers. These calculations were performed using the Monte Carlo method with an implemented MMFF94 force field [38]. The lowest-energy conformer was the initial structure in the later geometry optimization stage of the calculations. The geometries of all compounds were optimized using the density functional theory (DFT) method with the B3LYP/6-311G^∗∗^ hybrid functional implemented in the Spartan’20 software package [39]. The electrostatic potentials on a surface of equal electron density were calculated using the same functional and basic set as for the geometry optimization to determine the contribution of electron-donor and electron-acceptor sites into the electrostatic pattern of the molecule. The crystal structure of the BRAF_V600E_ kinase in a complex with vemurafenib (PLX-4032) (PDB accession code 3OG7) [35,40] was retrieved from Brookhaven Protein Data Bank (http://www.rcsb.org (accessed on 22 September 2010)). All ligands, inorganic ions, and solvent molecules that were present in the kinase original structure were manually removed, and hydrogen atoms were added using the graphical interface of DiscoveryStudio v.21.1. This complex structure consists of two homodimeric chains: A and B. Our goal was to target the mutated chain (chain A) of BRAF_V600E_; therefore, chain B was deleted from the structure of 3OG7.

### 3.2. Preliminary Molecular Docking

The molecular docking approach provides the most promising route for drug design and discovery. The ligand-binding pocket region of the BRAF_V600E_ kinase was selected as the binding site for screening the compounds that could potentially inhibit the BRAF_V600E_ molecule. For molecular docking, the as-designed compounds (VEM-1, VEM-2, VEM-3) and vemurafenib (reference BRAF_V600E_ inhibitor) were uploaded into the CDOCKER module in DiscoveryStudio v.21.1 visual interface BIOVIA [37], which relies on the CHARMm force field [41], providing docking results with high precision [42]. The binding site was defined with a radius of 18 Å around the ligand present in the X-ray structure of the protein BRAF_V600E_. The distinct conformational poses of each compound were generated and analyzed based on the CDOCKER interaction energy. The number of starting random conformations and several rotated ligand orientations to refine each of the conformations for 1000 dynamic steps was set to thirty. Moreover, for annealing refinement, the number of heating steps was 2000 and the number of cooling sets was set to 5000. The best-docked poses were selected, and then the molecular dynamic (MD) simulations were performed to improve the structural reliability of the ligand–BRAF_V600E_ complexes.

### 3.3. Molecular Dynamic Simulations and Binding Free Energy Calculations

MD simulation analysis was performed to find the interaction of ligand–protein stability. All MD simulations were run in the CHARMm force field implemented in the module of DiscoveryStudio v.21.1. Each complex was solvated in a TIP3P water cubic box, with periodic boundary conditions and a minimum distance of 1 Å from the surface of the complex to the edge of the box, and neutralized by adding Na^+^ and Cl^−^ ions to a physiological concentration of ~0.15 M [43]. Before simulations, 2500 steps of the steepest-descent algorithm followed by 5000 conjugate gradient energy-minimization steps (until the RMS gradient of the structure was below 0.01 kcal/mol Å) were performed. During minimization, the protein was restrained with a force constant of 10 kcal/mol Å^2^ and gradually decreased to 1 kcal/mol Å^2^. Subsequently, each simulation started with gradual heating from 50 to 300 K for 50 ps followed by equilibration of the systems up to 100 ps, after which potential energies were sufficient. The equilibrated system was taken as the starting structure for 100 ns production runs in the NPT (normal pressure and temperature) ensemble, at a temperature of 300 K and 1 bar maintained using a Berendsen thermostat algorithm [44]. In the stages of heating, equilibration, and production, the protein was restrained with a force constant of 1 kcal/mol Å^2^, but ligands and solvent molecules with counterions were allowed to move. The SHAKE method was used to constrain hydrogen atoms and the time step was set to 2 fs [45]. The coordinates were saved every 10 ps for subsequent analysis. The trajectories from the MD simulations were saved for every 50 ps interval for analyses of the root-mean-square deviation (RMSD) and the mean-square fluctuation (RMSF), as well as the protein–ligand contacts. The analysis of the interactions between the two molecules was performed based on the donor/acceptor distance given in Å.

The obtained stable MD trajectory of each complex was used to calculate the binding free energy by the MM-PBSA method (molecular mechanics Poisson–Boltzmann surface area) [46]. Every binding free energy value was calculated from the average structures obtained after a total of 501 frames had been extracted from the last 3 ns MD production trajectory at 4 ps intervals. The components of every complex were minimized using the conjugate gradient method for 10,000 steps, after 2000 steps of the steepest-descent algorithm, and a dielectric constant of 4 for the electrostatic interactions until the RMS gradient of the structure was <0.001 kcal/mol Å. The ΔG_bind_ was estimated, given the functional from of formalism as ΔG_bind_ = ΔG_BRAF-ligand_ − ΔG_BRAF_ − ΔG_ligand_, where ΔG_BRAF-ligand_ is the free energy of the complex, ΔG_BRAF_ is the free energy of the protein, and ΔG_ligand_ is the free energy of vemurafenib or its derivatives.

### 3.4. Computation of Physicochemical, Biopharmaceutical, and Toxicological Properties

The application of computational tools for identifying novel candidates such as BRAF inhibitors assists in lessening the number of experiments and increases the success rate [47]. We applied some drug-likeness rules as an initial screening step for oral bioavailability, followed by a secondary screening by calculating the ADME-TOX profile (absorption, distribution, metabolism, excretion, and toxicology) for a comprehensive measure of biodisposition and toxicological parameters [48] using the software ADMET Predictor^TM^ version 10.1 [49]. Three-dimensional structures of vemurafenib and its novel three derivatives (VEM-1, VEM-2, VEM-3) were exported into a 3D SDF format and introduced into the mathematical models implemented in the program with Lipinski’s rule of five, and the addition of the topological polar surface area (TPSA ≤ 140 Å^2^) was considered a standard for accessing the drug likeness. The next parameter was the volume of distribution (V_d_), which is the theoretical volume that relates the amount of drug in the body to the concentration of drug measured in a biological fluid, i.e., in the blood plasma. The V_d_ parameter should be ≤3.7 L/kg if the drug is distributed mainly in the blood plasma. The aqueous solubility (S_w_) was also calculated, which should be ≥0.010 mg/mL. More sophisticated in silico models for the oral dosage form were analyzed, including the effective permeability (P_eff_), which determines the rate and extent of intestinal drug absorption based on Fick’s law, for which values of ≥0.5 cm/s·10^−4^ are expected, and Madin–Darby canine kidney cell (MDCK) apparent permeability [50], which is a parameter for assessing the membrane permeability properties in early-stage drug discovery, for which values of ≥30 cm/s·10^−7^ are expected. The distribution profile was characterized by the percentage of unbound drug to proteins in plasma (PrUnbnd > 10%) and the blood-to-plasma concentration ratio (RBP < 1), which is another mode of expression of drug distribution within blood providing an indication of drug binding to erythrocytes, a qualitative likelihood of penetrating the blood–brain barrier (BBB filter expressed as high/low). The logarithm of the blood–brain barrier partition coefficient (logBB), which was more than 0.3 for high absorption, between 0.3 and −1.0 for middle absorption, and less than −1.0 for low absorption, was calculated. Prediction of metabolic phase I indicators was accomplished using various cytochrome P450 (CYP 450) isoforms. The metabolism module of the ADMET Predictor^TM^ includes five models for CYP isoforms (CYP1A2, 2C9, 2C19, 2D6 and 3A4), while the models for the substrate classification and atomic site of metabolism cover nine CYP isoforms (CYP1A2, 2A6, 2B6, 2C8, 2C9, 2C19, 2D6, 2E1, and 3A4). The likelihood of interactions with potential membrane transporters was assessed using six models to classify the mode of action (substrate or inhibition): P-glycoprotein (P-gp), the breast cancer resistance protein (BCRP), the hepatic organic anion transporting polypeptides (OATP1B1 and OATP1B3), the renal organic anion transporters (OAT1 and OAT3), the hepatic organic cation transporters (OCT1 and OCT2), and the bile salt export pump (BSEP). Biodisposition was also assessed on the basis of such parameters as the area under the curve (AUC), peak plasma concentration (C_max_), time to C_max_ (T_max_) and half-life at steady state (t_1/2_) [51]. These parameters were calculated using dose–response approaches on the strength of the advanced compartmental absorption and transit (ACAT^TM^) model, which consists of nine intestinal compartments and accounts for all relevant parameters that may impact oral drug absorption (physicochemical drug properties, formulation design, physiological conditions, and drug pharmacokinetic data) [52].

Virtual screening was also performed to assess the toxicity of vemurafenib and its new derivatives (VEM-1, VEM-2, VEM-3), including maximum recommended therapeutic dose (MRTD), affinity towards hERG-encoded potassium channel (hERG filter and hERG pIC_50_) associated with cardiac toxicity, and the levels of five hepatic enzymes (AlkPhos, GGT, LDH, AST, and ALT) used as hepatotoxicity biomarkers (DILI). Furthermore, several parameters linked to animal models were used to assess the systemic toxicity, such as the acute rat toxicity model (rat acute < 300 mg/kg) and two quantitative chronic carcinogenicity models (rat TD_50_ < 4 mg/kg day and mouse TD_50_ < 25 mg/kg day). Clastogenic and mutagenic (MUT) studies were also performed based on computing the chromosome aberration (Chrom Aberr) and *Salmonella typhimurium*, depicting the results of virtual AMES testing. Ten MUT models were used, which were used individually in the assessment of the mutagenicity anticipated for five strains of *Salmonella typhimurium* with microsomal activation (MUT_m97+1537_; MUT_m98_; MUT_m100_; MUT_m102+wp2_ and MUT_m1535_) and without microsomal activation (MUT_97+1537_; MUT_98_; MUT_100_; MUT_102+wp2_ and MUT_1535_). The final toxicity profile of the tested compounds was based on the TOX_Risk_ model, which includes seven rules (potential hERG liability, acute toxicity in rats, carcinogenicity in chronic rat studies, carcinogenicity in chronic mouse studies, and hepatotoxicity and SGOT and SGPT evaluation), each of which has an associated weight of one. Possible value ranges of 0–11 for MUT_Risk_ and 0–7 for TOX_Risk_ were accepted.

## 4. Conclusions

The BRAF_V600E_ serine-threonine kinase is a vital and attractive therapeutic target in melanoma and other types of cancers. However, acquired resistance to treatment with BRAF V600E kinase inhibitors, such as vemurafenib, and the side effects of some other drugs used in BRAF-mutated melanoma cause several clinical problems [53]. Considering these facts, we designed new vemurafenib derivatives that contain a carbohydrate moiety and attempted to investigate their biopharmaceutical, pharmacokinetic (ADME) and toxicological properties, as well as the mechanism of interaction with BRAF_V600E_ kinase based on molecular modeling using in silico approaches.

The tested series of the compounds containing methyl-, acetyl-, and isopropyl-ketopentose moieties showed that further expansion of the acyl substituent over isopropyl in the pentose is not justified and has no further effect on predicted activity. All tested compounds are predicted to be able to inhibit the serine-threonine kinase by totally occupying the active site of the mutated BRAF_V600E_ kinase. Nevertheless, the newly designed vemurafenib analogue VEM-3 showed better binding affinity towards BRAF_V600E_ kinase than the reference inhibitor (vemurafenib), showing a common molecular interaction with residues Leu-505, Ile-527, Cys-532, Gly-534, Gly-593, Asp-594 and Gly-696 of the BRAF_V600E_ oncoprotein. The stability of the complex might also be associated with extra alkyl interaction with Arg-509 and Leu-515, π–alkyl interaction with four residues (Val-471, Ala-481, Lys-483 and Leu-505), and lastly π–cation interaction between the pyrrole ring of VEM-3 and Lys-483 residue. Additionally, the isopropyl-ketopentose substituent of VEM-3 creates the next hydrogen bond with residues Gly-534 and Ser-536 and two water molecules. Moreover, based on the evaluation of ADME and the toxicity profile of the potential lead compound (VEM-3), which does not violate the drug-likeness rules and has lower toxic effects, we can assume that it is the most suitable candidate for further analysis.

Overall, it can be concluded that the use of molecular docking, ADMET analysis, protein–ligand binding analysis, and dynamic simulation revealed that the as-designed vemurafenib derivative (VEM-3) should be synthesized and subjected to further in vitro and in vivo studies, which may be able to open a new avenue to next-generation BRAF_V600E_ kinase inhibitors to treat metastatic melanoma.

## Figures and Tables

**Table 1:** Theoretical values of water solubility (S_w_), effective permeability (P_eff_), apparent permeability (MDCK), percentage of the unbound drug to blood plasma proteins (%Unbnd), blood-to-plasma concentration ratio (RBP), blood–brain barrier filter (BBB), and blood–brain barrier partition coefficient (logBB) for vemurafenib and VEM-1, VEM-2 and VEM-3 derivatives.

Compound	V_d_	S_w_	P_eff_	MDCK	%Unbnd	RBP	BBB Filter	logBB
	Expected Values							
	(≤3.7 L/kg)	(≥0.010 mg/mL)	(≥0.5 cm/s·10^−4^)	(≥30 cm/s·10^−7^)	(>10%)	(<1)	(High/Low)	
vemurafenib	1.54	0.0000006	1.683	135.93	2.60	0.72	Low	−0.152
VEM-1	1.02	0.00013	0.337	12.62	4.77	0.69	Low	−1.157
VEM-2	1.05	0.00011	0.284	13.89	4.06	0.68	Low	−1.103
VEM-3	1.10	0.000092	0.241	14.22	3.86	0.68	Low	−1.011

**Table 2:** Mode of action (inhibition/substrate) of selected transporters (P-glycoprotein (P-gp), breast cancer resistance protein (BCRP), bile salt export pump (BSEP), and hepatic transporters (OATP1B1 and OATP1B3)) for vemurafenib and its derivatives (VEM-1, VEM-2 and VEM-3).

Compound	P-gp Substrate/ Inhibitor	Bcrp Substrate/ Inhibitor	BSEP Inhibitor	BSEP_IC_50_	OATP1B1 Inhibitor	OATP1B3 Inhibitor
				(≤60 μM)		
vemurafenib	No	Yes	Yes	22.67	Yes	No
VEM-1	Yes	Yes	Yes	11.44	Yes	Yes
VEM-2	Yes	Yes	Yes	9.80	Yes	Yes
VEM-3	Yes	Yes	Yes	10.07	Yes	Yes

**Table 3:** Predicted human toxicity parameters for vemurafenib and is derivatives (VEM-1, VEM-2, VEM-3): maximum recommended therapeutic dose (MRTD); cardiotoxicity—the hERG filter and affinity for hERG K^+^ (hERG pIC_50_); human liver adverse effect (the likelihood of causing an elevation in the levels of alkaline phosphatase (AlkPhos), γ-glutamyl transferase (GGT), lactate dehydrogenase (LDH), serum glutamate oxaloacetate transaminase (SGOT) and serum glutamate pyruvate transaminase (SGPT)).

Compound	MRTD	hERG Filter	hERG pIC_50_	AlkPhos	GGT	LDH	SGOT
	Expected Values						
	(>3.16 mg/kg/day)	(Yes/No)	(>5.5)				
vemurafenib	Below 3.16	Yes	5.06	T	NT	NT	NT
VEM-1	Below 3.16	No	4.38	T	NT	NT	NT
VEM-2	Below 3.16	No	4.36	T	NT	NT	NT
VEM-3	Below 3.16	No	4.33	T	NT	NT	NT

**Table 4:** Predicted toxicity parameters employing in silico animal models for vemurafenib and its derivatives (VEM-1, VEM-2 and VEM-3): carcinogenicity (TD_50_) in mouse and rat; LD_50_ for lethal oral acute toxicity in rat and the qualitative estimation of the risk of clastogenic (chromosomal aberrations) and mutagenic (AMES) assays studied with and without rat liver microsomal activation.

Compound	Mouse TD_50_	Rat TD_50_	Rat Acute LD_50_	Chrom_Aberr	MUT_97+1537_	MUT^(*)^
	Expected Values					
	(<25 mg/kg/day)	(<4 mg/kg/day)	(<300 mg/kg)			
vemurafenib	623.15	21.93	44.0	NT	Yes	No
VEM-1	628.50	1.99	135.98	NT	No	No
VEM-2	669.95	1.70	140.63	NT	Yes	No
VEM-3	670.56	1.38	156.29	NT	Yes	No

MUT^(*)^—classification model for the mutagenicity of pure compounds in: MUT_98_; MUT_100_; MUT_102+wp2_; MUT_1535_ and mutagenicity of microsomal rat liver metabolites in: MUT_m97+1537_; MUT_m98_; MUT_m100_; MUT_m102+wp2_; MUT_m1535_.

## Data Availability

All relevant data are provided in the article.

## Supplementary Materials

The following supporting information can be downloaded at https://www.mdpi.com/article/10.3390/molecules28135273/s1. Figure S1: Simulated plasma concentration–time profiles in humans after oral administration of vemurafenib (A), VEM-1 (B), VEM-2 (C), and VEM-3 (D) at doses of 240 mg, 480 mg, and 960 mg. Figure S2: Simulated and crystal structure of the BRAF_V600E_–vemurafenib complex. (A) Comparison of the position between the docked structure (yellow) and the X-ray crystal structure (green) of vemurafenib in the ATP-binding site. The binding mode of vemurafenib (reference molecule) in the crystal structure of BRAF kinase (B) and vemurafenib in the structure of BRAF kinase after docking (C). Table S1: The topological polar surface area (TPSA) and parameters of Lipinski’s rule of five for vemurafenib and its derivatives (VEM-1, VEM-2, VEM-3). Table S2: Mode of action (inhibition/substrate) and intrinsic hepatic clearance (Cl_int_) for selected cytochrome P450 isoforms for vemurafenib and its derivatives (VEM-1, VEM-2, VEM-3). Table S3: Mode of action (inhibition/substrate) of selected transporter models (likelihood of the renal organic anion transporters (OAT1 and OAT3) and hepatic organic cation transporters (OCT1 and OCT2)) for vemurafenib and its derivatives (VEM-1, VEM-2, VEM-3). Table S4: Comparison between predicted and observed pharmacokinetic parameters of vemurafenib administered at three different doses. Table S5: Predicted pharmacokinetic parameters for VEM-1, VEM-2 and VEM-3 administered at three different doses. Table S6: Toxicity risk for vemurafenib and its derivatives (VEM-1, VEM-2, VEM-3) employing mutagenicity models (in silico AMES test). Table S7: Intermolecular interactions of vemurafenib and its derivatives (VEM-1, VEM-2, VEM-3) in the active site of the BRAF_V600E_ kinase.

## References

[1] Bonner T., O’Brien S.J., Nash W.G., Rapp U.R., Morton C.C., Leder P. (1984). The human homologs of the raf (mil) oncogene are located on human chromosomes 3 and 4. Science.

[2] Molina J.R., Adjei A.A. (2006). The Ras/Raf/MAPK pathway. J. Thorac. Oncol..

[3] Wan P.T., Garnett M.J., Roe S.M., Lee S., Niculescu-Duvaz D., Good V.M., Jones C.M., Marshall C.J., Springer C.J., Barford D. (2004). Mechanism of activation of the RAF-ERK signaling pathway by oncogenic mutations of B-RAF. Cell.

[4] Davies H., Bignell G.R., Cox C., Stephens P., Edkins S., Clegg S., Teague J., Woffendin H., Garnett M.J., Bottomley W. (2002). Mutations of the BRAF gene in human cancer. Nature.

[5] Flaherty K.T., Puzanov I., Kim K.B., Ribas A., McArthur G.A., Sosman J.A., O’Dwyer P.J., Lee R.J., Grippo J.F., Nolop K. (2010). Inhibition of mutated, activated BRAF in metastatic melanoma. N. Engl. J. Med..

[6] Swaika A., Crozier J.A., Joseph R.W. (2014). Vemurafenib: An evidence-based review of its clinical utility in the treatment of metastatic melanoma. Drug Des. Dev. Ther..

[7] Poulikakos P.I., Zhang C., Bollag G., Shokat K.M., Rosen N. (2010). RAF inhibitors transactivate RAF dimers and ERK signalling in cells with wild-type BRAF. Nature.

[8] Bollag G., Tsai J., Zhang J., Zhang C., Ibrahim P., Nolop K., Hirth P. (2012). Vemurafenib: The first drug approved for BRAF-mutant cancer. Nat. Rev. Drug Discov..

[9] Halaban R., Zhang W., Bacchiocchi A., Cheng E., Parisi F., Ariyan S., Krauthammer M., McCusker J.P., Kluger Y., Sznol M. (2010). PLX4032, a selective BRAF(V600E) kinase inhibitor, activates the ERK pathway and enhances cell migration and proliferation of BRAF melanoma cells. Pigment. Cell Melanoma Res..

[10] Tsai J., Lee J.T., Wang W., Zhang J., Cho H., Mamo S., Bremer R., Gillette S., Kong J., Haass N.K. (2008). Discovery of a selective inhibitor of oncogenic B-Raf kinase with potent antimelanoma activity. Proc. Natl. Acad. Sci. USA.

[11] Smalley K.S., Lioni M., Dalla Palma M., Xiao M., Desai B., Egyhazi S., Hansson J., Wu H., King A.J., Van Belle P. (2008). Increased cyclin D1 expression can mediate BRAF inhibitor resistance in BRAF V600E-mutated melanomas. Mol. Cancer Ther..

[12] Johnson D.B., Menzies A.M., Zimmer L., Eroglu Z., Ye F., Zhao S., Rizos H., Sucker A., Scolyer R.A., Gutzmer R. (2015). Acquired BRAF inhibitor resistance: A multicenter meta-analysis of the spectrum and frequencies, clinical behaviour, and phenotypic associations of resistance mechanisms. Eur. J. Cancer.

[13] Shaffer S.M., Dunagin M.C., Torborg S.R., Torre E.A., Emert B., Krepler C., Beqiri M., Sproesser K., Brafford P.A., Xiao M. (2017). Rare cell variability and drug-induced reprogramming as a mode of cancer drug resistance. Nature.

[14] Ji Z., Erin Chen Y., Kumar R., Taylor M., Jenny Njauw C.N., Miao B., Frederick D.T., Wargo J.A., Flaherty K.T., Jonsson G. (2015). MITF Modulates Therapeutic Resistance through EGFR Signaling. J. Investig. Dermatol..

[15] Hu W., Jin L., Jiang C.C., Long G.V., Scolyer R.A., Wu Q., Zhang X.D., Mei Y., Wu M. (2013). AEBP1 upregulation confers acquired resistance to BRAF (V600E) inhibition in melanoma. Cell Death Dis..

[16] Villanueva J., Vultur A., Lee J.T., Somasundaram R., Fukunaga-Kalabis M., Cipolla A.K., Wubbenhorst B., Xu X., Gimotty P.A., Kee D. (2010). Acquired resistance to BRAF inhibitors mediated by a RAF kinase switch in melanoma can be overcome by cotargeting MEK and IGF-1R/PI3K. Cancer Cell.

[17] Das Thakur M., Salangsang F., Landman A.S., Sellers W.R., Pryer N.K., Levesque M.P., Dummer R., McMahon M., Stuart D.D. (2013). Modelling vemurafenib resistance in melanoma reveals a strategy to forestall drug resistance. Nature.

[18] Flaherty K.T., Infante J.R., Daud A., Gonzalez R., Kefford R.F., Sosman J., Hamid O., Schuchter L., Cebon J., Ibrahim N. (2012). Combined BRAF and MEK inhibition in melanoma with BRAF V600 mutations. N. Engl. J. Med..

[19] Pelster M.S., Amaria R.N. (2019). Combined targeted therapy and immunotherapy in melanoma: A review of the impact on the tumor microenvironment and outcomes of early clinical trials. Ther. Adv. Med. Oncol..

[20] Rager T., Eckburg A., Patel M., Qiu R., Gantiwala S., Dovalovsky K., Fan K., Lam K., Roesler C., Rastogi A. (2022). Treatment of Metastatic Melanoma with a Combination of Immunotherapies and Molecularly Targeted Therapies. Cancers.

[21] Froelich W. (2020). New BRAF inhibitor shows promise in a variety of cancers. Oncol. Times.

[22] Tutuka C.S.A., Andrews M.C., Mariadason J.M., Ioannidis P., Hudson C., Cebon J., Behren A. (2017). PLX8394, a new generation BRAF inhibitor, selectively inhibits BRAF in colonic adenocarcinoma cells and prevents paradoxical MAPK pathway activation. Mol. Cancer.

[23] Sumardika I.W., Youyi C., Kondo E., Inoue Y., Ruma I.M.W., Murata H., Kinoshita R., Yamamoto K.I., Tomida S., Shien K. (2018). beta-1,3-Galactosyl-O-Glycosyl-Glycoprotein beta-1,6-N-Acetylglucosaminyltransferase 3 Increases MCAM Stability, Which Enhances S100A8/A9-Mediated Cancer Motility. Oncol. Res..

[24] Very N., Lefebvre T., El Yazidi-Belkoura I. (2018). Drug resistance related to aberrant glycosylation in colorectal cancer. Oncotarget.

[25] Kudo T., Nakagawa H., Takahashi M., Hamaguchi J., Kamiyama N., Yokoo H., Nakanishi K., Nakagawa T., Kamiyama T., Deguchi K. (2007). N-glycan alterations are associated with drug resistance in human hepatocellular carcinoma. Mol. Cancer.

[26] De Vellis C., Pietrobono S., Stecca B. (2021). The Role of Glycosylation in Melanoma Progression. Cells.

[27] Nisiewicz M.K., Kowalczyk A., Sobiepanek A., Jagielska A., Wagner B., Nowakowska J., Gniadek M., Grudzinski I.P., Kobiela T., Nowicka A.M. (2021). Tracking of Glycans Structure and Metallomics Profiles in BRAF Mutated Melanoma Cells Treated with Vemurafenib. Int. J. Mol. Sci..

[28] Tang L., Chen X., Zhang X., Guo Y., Su J., Zhang J., Peng C., Chen X. (2019). N-Glycosylation in progression of skin cancer. Med. Oncol..

[29] Lipinski C.A., Lombardo F., Dominy B.W., Feeney P.J. (2001). Experimental and computational approaches to estimate solubility and permeability in drug discovery and development settings. Adv. Drug Deliv. Rev..

[30] Ntie-Kang F. (2013). An in silico evaluation of the ADMET profile of the StreptomeDB database. Springerplus.

[31] Durmus S., Sparidans R.W., Wagenaar E., Beijnen J.H., Schinkel A.H. (2012). Oral availability and brain penetration of the B-RAFV600E inhibitor vemurafenib can be enhanced by the P-GLYCOprotein (ABCB1) and breast cancer resistance protein (ABCG2) inhibitor elacridar. Mol. Pharm..

[32] Thapar M.M., Ashton M., Lindegardh N., Bergqvist Y., Nivelius S., Johansson I., Bjorkman A. (2002). Time-dependent pharmacokinetics and drug metabolism of atovaquone plus proguanil (Malarone) when taken as chemoprophylaxis. Eur. J. Clin. Pharmacol..

[33] Goldinger S.M., Rinderknecht J., Dummer R., Kuhn F.P., Yang K.H., Lee L., Ayala R.C., Racha J., Geng W., Moore D. (2015). A single-dose mass balance and metabolite-profiling study of vemurafenib in patients with metastatic melanoma. Pharmacol. Res. Perspect..

[34] Zhang W., Heinzmann D., Grippo J.F. (2017). Clinical Pharmacokinetics of Vemurafenib. Clin. Pharmacokinet..

[35] Bollag G., Hirth P., Tsai J., Zhang J., Ibrahim P.N., Cho H., Spevak W., Zhang C., Zhang Y., Habets G. (2010). Clinical efficacy of a RAF inhibitor needs broad target blockade in BRAF-mutant melanoma. Nature.

[36] Sargsyan K., Grauffel C., Lim C. (2017). How Molecular Size Impacts RMSD Applications in Molecular Dynamics Simulations. J. Chem. Theory Comput..

[37] Dassault Systèmes BIOVIA (2016). Discovery Studio Modeling Environment, Release 2017.

[38] Halgren T.A. (1996). Merck molecular force field 3. Molecular geometries and vibrational frequencies for MMFF94. J. Comput. Chem..

[39] Hehre W., Ohlinger S. (2022). Spartan 20 for Windows, Machintosh and Linux Tutorial and Users ’Guide.

[40] Brose M.S., Volpe P., Feldman M., Kumar M., Rishi I., Gerrero R., Einhorn E., Herlyn M., Minna J., Nicholson A. (2002). BRAF and RAS mutations in human lung cancer and melanoma. Cancer Res..

[41] Brooks B.R., Brooks C.L., Mackerell A.D., Nilsson L., Petrella R.J., Roux B., Won Y., Archontis G., Bartels C., Boresch S. (2009). CHARMM: The biomolecular simulation program. J. Comput. Chem..

[42] Gagnon J.K., Law S.M., Brooks C.L. (2016). Flexible CDOCKER: Development and application of a pseudo-explicit structure-based docking method within CHARMM. J. Comput. Chem..

[43] Jorgensen W.L., Chandrasekhar J., Madura J.D., Impey R.W., Klein M.L. (1983). Comparison of Simple Potential Functions for Simulating Liquid Water. J. Chem. Phys..

[44] Berendsen H.J.C., Postma J.P.M., Vangunsteren W.F., Dinola A., Haak J.R. (1984). Molecular-Dynamics with Coupling to an External Bath. J. Chem. Phys..

[45] Ryckaert J.P., Ciccotti G., Berendsen H.J.C. (1977). Numerical integration of the cartesian equations of motion of a system with constraints: Molecular dynamics of n-alkanes. J. Comput. Phys..

[46] Kollman P.A., Massova I., Reyes C., Kuhn B., Huo S., Chong L., Lee M., Lee T., Duan Y., Wang W. (2000). Calculating structures and free energies of complex molecules: Combining molecular mechanics and continuum models. Acc Chem. Res..

[47] Chandrasekaran B., Abed S.N., Al-Attraqchi O., Kuche K., Tekade R.K. (2018). Computer-Aided Prediction of Pharmacokinetic (ADMET) Properties.

[48] Martinez-Mayorga K., Madariaga-Mazon A., Medina-Franco J.L., Maggiora G. (2020). The impact of chemoinformatics on drug discovery in the pharmaceutical industry. Expert Opin. Drug Discov..

[49] (2020). ADMET Predictor.

[50] Veber D.F., Johnson S.R., Cheng H.Y., Smith B.R., Ward K.W., Kopple K.D. (2002). Molecular properties that influence the oral bioavailability of drug candidates. J. Med. Chem..

[51] Hosea N.A., Jones H.M. (2013). Predicting pharmacokinetic profiles using in silico derived parameters. Mol. Pharm..

[52] Agoram B., Woltosz W.S., Bolger M.B. (2001). Predicting the impact of physiological and biochemical processes on oral drug bioavailability. Adv. Drug Deliv. Rev..

[53] Savoia P., Zavattaro E., Cremona O. (2020). Clinical Implications of Acquired BRAF Inhibitors Resistance in Melanoma. Int. J. Mol. Sci..

