# Supplementary material for: In Silico Studies of Novel Vemurafenib Derivatives as BRAF Kinase Inhibitors
Source: Molecules. 2023 Jul 7;28(13):5273. doi: 10.3390/molecules28135273 (PMC10343629; doi:10.3390/molecules28135273)
Supplement: Supplementary file 1 [file molecules-28-05273-s001.zip › molecules-2399584-supplementary.pdf]

molecules

Supplementary Material

Table S1. The topological polar surface area (TPSA) and parameters of Lipinski´s rule of five for vemurafenib and its derivatives (VEM-1, VEM-2, VEM-3).

Compound	TPSA	MWt	LogP	HBD	HBA
	expected values				
	(<140Å^2)	(≤500 g mol^{-1})	(≤5)	(≤5)	(≤8)
vemurafenib	91.92	489.93	5.17	2	5
VEM-1	177.14	645.64	3.26	4	11
VEM-2	177.14	659.67	3.59	4	11
VEM-3	177.14	673.69	3.96	4	11

Table S2. Mode of action (inhibition/substrate) and intrinsic hepatic clearance (Cl_{int}) for selected cytochrome P450 isoforms for vemurafenib and its derivatives (VEM-1, VEM-2, VEM-3).

Compound	CYP					
	Mode of action			C_{lint} (µL/min/mg)		
	1A2	2C9	3A4	1A2	2C9	3A4
vemurafenib	S/I	S	S	4.84	220.15	76.64
VEM-1	S/I	NS	S	15.26	-	1238.95
VEM-2	S/I	NS	S/I	13.97	-	903.74
VEM-3	S/I	NS	S/I	10.38	-	832.65

I-indicates inhibition of CYP isoforms; S-indicates substrate for CYP isoforms; NS-indicates non substrate for isoforms.

Table S3. Mode of action (inhibition/substrate) of selected transporters models (likelihood of the renal organic anion transporters (OAT1 and OAT3) and hepatic organic cation transporters (OCT1 and OCT2)) for vemurafenib and its derivatives (VEM-1, VEM-2, VEM-3).

Compound	OAT1 substrate/ inhibitor	OAT3 substrate/ inhibitor	OCT1 substrate/ inhibitor	OCT2 substrate/ inhibitor
vemurafenib	No/Yes	No/Yes	No/Yes	No/Yes
VEM-1	No/Yes	No/Yes	No/Yes	No/Yes
VEM-2	No/Yes	No/Yes	No/Yes	No/Yes
VEM-3	No/Yes	No/Yes	No/Yes	No/Yes

Table S4. The comparison between predicted and observed pharmacokinetic parameters of vemurafenib administered at three different doses.

Parameters	Vemurafenib					
	Predicted			Observed [1]		
Dose [mg]	240 mg	480 mg	960 mg	240 mg	480 mg	960 mg
$C_{max\ 0\text{-}8h}$ [ng/mL]	1.1	2.1	3.8	1.9	2.6	4.8
$T_{max\ 0\text{-}8h}$ [h]	5.0	5.0	5.9	4.0	4.0	5.0
$AUC_{0\text{-}8h}$ [ng-h/mL]	6.2	11.8	21.3	8.3	13.8	27.0
$AUC_{0\text{-}24h}$ [ng-h/mL]	18.4	35.0	63.3	40.9	62.4	130.6
$t_{1/2}$ [h]	15.1	15.0	15.0	31.5	38.4	34.1

Table S5. Predicted pharmacokinetic parameters for VEM-1, VEM-2 and VEM-3 administered at three different doses.

Parameters	VEM-1			VEM-2			VEM-3		
Dose [mg]	240 mg	480 mg	960 mg	240 mg	480 mg	960 mg	240 mg	480 mg	960 mg
$C_{max\ 0\text{-}8h}$ [ng/mL]	13.9	19.1	23.6	6.1	8.1	9.7	3.0	3.6	4.2
$T_{max\ 0\text{-}8h}$ [h]	5.8	5.8	6.0	6.3	6.3	6.5	6.3	6.4	6.6
$AUC_{0\text{-}8h}$ [ng-h/mL]	80.0	110.0	135.3	34.5	45.5	54.3	16.6	20.2	23.7
$AUC_{0\text{-}24h}$ [ng-h/mL]	230.3	316.9	393.0	107.6	141.7	170.1	51.7	62.7	73.45
$t_{1/2}$ [h]	14.0	13.9	13.8	17.5	17.3	17.2	16.7	16.5	16.38

Table S6. Toxicity risk for vemurafenib and its derivatives (VEM-1, VEM-2, VEM-3) employing mutagenicity models (*in silico* AMES test).

Compound	MUT_{Risk}	MUT_{xRisk}	TOX_{Risk}	MUT_{Code}
	expected values			
	(>2)	(0-4)	(0-7)	
vemurafenib	0.6	0.35	1	S_97
VEM-1	0	0	1.5	-
VEM-2	0.6	0.35	1.5	S_97
VEM-3	0.6	0.35	2	S_97

Filters provide a qualitative estimate of potential toxicity concerns. Approximately 16% of commercial drugs within the focused subset of the WDI receive a MUT_{Risk} score > 1 and approximately 4% have a score > 2. TOX_{Risk} evaluates overall toxicological concerns and is greater than 3.3 for ~10% of the focused WDI.

Table S7. Intermolecular interactions of vemurafenib and its derivatives (VEM-1, VEM-2, VEM-3) in the active site of the $BRAF_{V600E}$ kinase.

Compound	Hydrogen bond	Distance H-A [Å]	Hydrophobic interaction	Distance [Å]	Electrostatic and halogen interactions	Distance [Å]
Vemurafenib	Cys-532	2.93	His-539	5.09	Lys-483	2.87
	Gln-530	2.88	Trp-531	4.63; 5.72; 5.83	Ala-481	3.57
	Asp-594	2.14; 2.21	Phe-583	4.94		
	Gly-569	3.36	Leu-514	5.09		
	Phe-595	3.13	Leu-505	4.24		
	Ser-535	2.92				
VEM-1	Cys-532	3.25	Val-471	5.02; 5.23	Phe-596	3.63
	Gly-534	2.60	Ala-481	4.33	Cys-532	5.63
	Ile-527	3.14	Leu-567	5.34	Asp-594	3.65
	Lys-483	2.19	Ile-572	4.18		
	Asp-594	2.96	Lys-507	4.88		
	Gly-593	3.13	Phe-595	5.30; 5.69		
	Gly-596	3.08				
	Leu-505	2.97				
	Phe-595	2.98				
	TIPW-3748	3.80				
	TIPW-5244	2.42				
	TIPW-1760	2.01				
	TIPW-2605	2.25				
VEM-2	Asp-594	2.77	Phe-583	4.11	-	-
	Gly-596	3.28	Ala-481	4.26; 4.37		
	Phe-595	3.26	Val-471	5.01; 5.29		
	Thr-529	3.98; 3.65	Lys-483	3.63; 4.12		
	TIPW-4050	2.60	Leu-515	4.74		
	TIPW-2420	2.25	Phe-595	4.64		
	TIPW-4803	2.27				
	TIPW-1538	2.05				
VEM-3	Cys-532	2.85; 2.35	Phe-583	4.44	Lys-483	4.57
	Ile-527	2.82	Trp-531	5.63	Leu-514	3.12
	Gly-596	3.09	Ala-481	4.48; 4.43		
	Phe-595	2.97	Val-471	5.13; 5.24		
	Thr-508	1.52	Lys-483	3.78; 3.94		
	Leu-514	1.77	Leu-515	5.28		
	Asp-594	2.81	Arg-509	4.28		
	Leu-505	2.84; 2.46	Phe-595	4.22		
	Gly-593	3.03				
	Ser-536	3.40				

	Gly-534	3.60
	Arg-603	2.99
	TIPW-1936	2.87
	TIPW-990	3.60

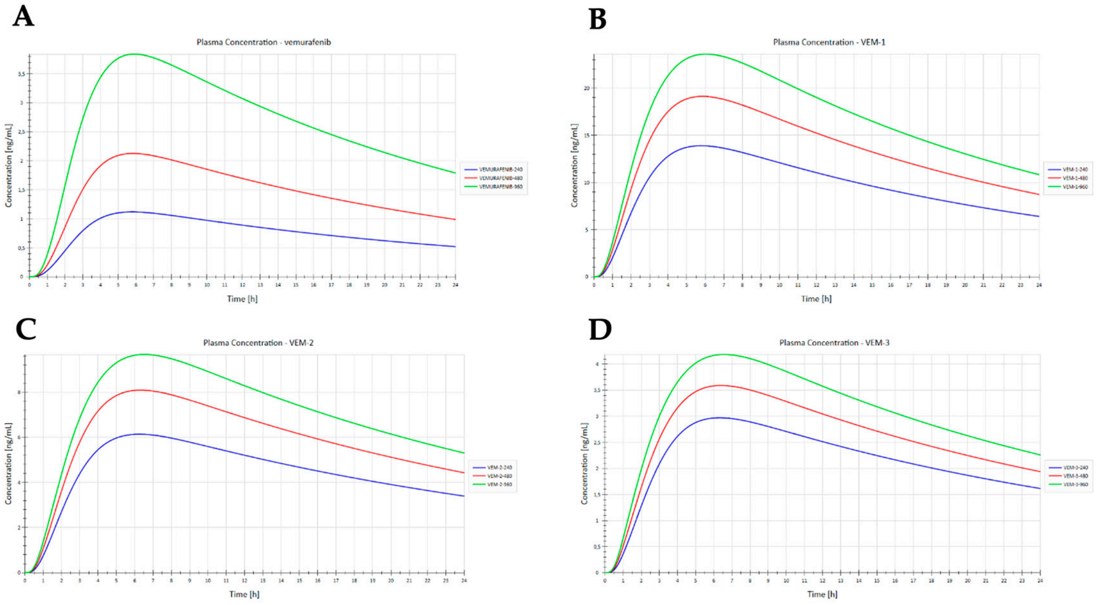

Figure S1. Simulated plasma concentration-time profiles in humans after oral administration of vemurafenib (A) and VEM-1 (B), VEM-2 (C) and VEM-3 (D) at doses of 240 mg, 480 mg and 960 mg.

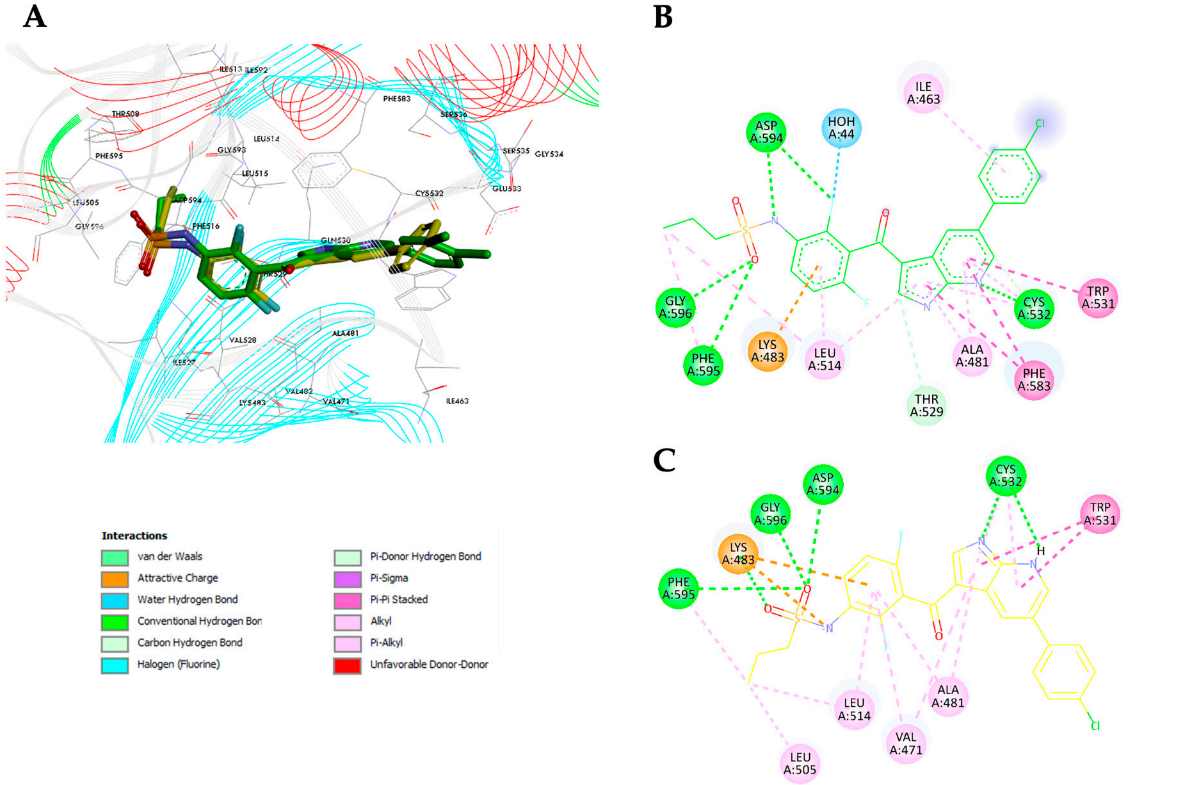

Figure S2. Simulated and crystal structure of the $BRAF_{V600E}$-vemurafenib complex. (A) Comparison of the position between the docked structure (yellow color) and the X-ray crystal structure (green color) of vemurafenib in the ATP binding site. The binding mode of vemurafenib (reference molecule) in the crystal structure of BRAF kinase (B), and vemurafenib in the structure of BRAF kinase after docking (C).

Reference

1. Zhang, W.; Heinzmann, D.; Grippo, J.F. Clinical Pharmacokinetics of Vemurafenib. *Clin Pharmacokinet* **2017**, *56*, 1033-1043, doi:10.1007/s40262-017-0523-7.
